# Variations in Well-Being as a Function of Paranormal Belief and Psychopathological Symptoms: A Latent Profile Analysis

**DOI:** 10.3389/fpsyg.2022.886369

**Published:** 2022-06-24

**Authors:** Neil Dagnall, Andrew Denovan, Kenneth Graham Drinkwater

**Affiliations:** ^1^Department of Psychology, Manchester Metropolitan University, Manchester, United Kingdom; ^2^Department of Psychology, University of Huddersfield, Huddersfield, United Kingdom

**Keywords:** paranormal belief, psychopathology, well-being, latent profile analysis, schizotypy

## Abstract

This study examined variations in well-being as a function of the interaction between paranormal belief and psychopathology-related constructs. A United Kingdom-based, general sample of 4,402 respondents completed self-report measures assessing paranormal belief, psychopathology (schizotypy, depression, manic experience, and depressive experience), and well-being (perceived stress, somatic complaints, and life satisfaction). Latent profile analysis identified four distinct sub-groups: Profile 1, high Paranormal Belief and Psychopathology (*n* = 688); Profile 2, high Paranormal Belief and Unusual Experiences; moderate Psychopathology (*n* = 800); Profile 3, moderate Paranormal Belief and Psychopathology (*n* = 846); and Profile 4, low Paranormal Belief and Psychopathology (*n* = 2070). Multivariate analysis of variance (MANOVA) found that sub-groups with higher psychopathology scores (Profiles 1 and 3) reported lower well-being. Higher Paranormal Belief, however, was not necessarily associated with lower psychological adjustment and reduced well-being (Profile 2). These outcomes indicated that belief in the paranormal is not necessarily non-adaptive, and that further research is required to identify the conditions under which belief in the paranormal is maladaptive.

## Introduction

Historically, studies have reported positive correlations between belief in the paranormal and psychopathological outcomes (Thalbourne and Storm, 2019; Liu et al., 2021). These include, but are not restricted to, greater incidence of psychiatric (Dag, 1999; Peltzer, 2002), depressive (Thalbourne and French, 1995), and manic (Thalbourne and French, 1995) symptoms. The importance of paranormal belief in relation to well-being is demonstrated by superstition, which is a specific facet of supernatural credence that indexes the notion that forces such as luck and fate influence real-world events (Dagnall et al., 2007b,^2009^). Superstition is associated with poorer psychological adjustment (see Wiseman and Watt, 2004; Dagnall et al., 2007b,^2009^; Liu et al., 2021). Particularly, negative features such as lower self-efficacy (Tobacyk and Shrader, 1991), greater anxiety (Wolfradt, 1997), higher neuroticism (Vyse, 2013), and external locus of control (Dag, 1999) (Hoffmann et al., 2022). Collectively, these findings imply direct relationships between paranormal belief, psychopathology, and well-being (Irwin, 2009).

A commonly cited explanation for these associations is the psychodynamic functions hypothesis (Irwin, 2009). This proposes that paranormal belief arises from personal attempts to impose order on the world. Belief in this context resolves uncertainty by providing meaning and/or the illusion of control (Irwin, 1993, 2003, 2009). Central to this process is magical ideation, which denotes “belief in forms of causation that by conventional standards are invalid” (Eckblad and Chapman, 1983, p. 215). Magical ideation often functions as a coping strategy when individuals believe they lack power (Ofori et al., 2017; Drinkwater et al., 2019). Consistent with this supposition, McGarry and Newberry (1981) reported that paranormal believers have a generalised tendency to view the world as unjust, problematic, and unpredictable (Roe and Bell, 2016; Stone, 2016).

The notion that paranormal belief can in some circumstances provide a sense of control implies that credence performs an adaptive function (Schumaker, 1987; Dean et al., 2021; Parra and Giudici, 2022). This, however, this is not necessarily the case since psychological benefits are typically restricted to specific situations (Roe and Bell, 2016). Thus, paranormal belief generally signifies poorer psychological functioning and is indicative of heightened distress. Though there is scholarly evidence to support this proposition, it is inconsistent with the high levels of paranormal endorsement (credence, experience, and ability) observed in non-clinical populations (see Dagnall et al., 2016c; Williams et al., 2021). This is evidenced by surveys, which report that paranormal belief is common within contemporary Western societies. For instance, a 2005 Gallup poll (Moore, 2005) found that three in four Americans acknowledged at least one paranormal belief (Irwin et al., 2012a).

Recognising the prevalence of paranormal beliefs, it is reasonable to conclude that within general samples supernatural credence, in the absence of concomitant cognitive-perceptual characteristics, has a benign (non-threatening) effect on well-being. Thus, paranormal belief is only problematic when it interacts with psychological factors, which distort perception and mentation (Irwin et al., 2012a,b). In such instances, paranormal belief may serve as an interpretative lens that structures cognitions (Drinkwater et al., 2021). This supposition suggests that supernatural credence is indicative, not determinative of mental state. From this perspective, paranormal belief has only an indirect effect on well-being *via* its associations with cognitive-perceptual factors.

### The Role of Schizotypy

A potential cognitive-perceptual catalyst is schizotypy (Dagnall et al., 2010, 2017a). Researchers have consistently reported a positive correlation between paranormal belief and schizotypy (see Dagnall et al., 2017a). This association is principally attributable to the positive characteristics of schizotypy (i.e., odd beliefs, unusual perceptual experiences, negative affect, and affective dysregulation; Barrantes-Vidal et al., 2013), which are likely to promote supernatural credence. Corresponding with this presumption, paranormal belief is mostly strongly correlated to the cognitive-perceptual factor of the Schizotypal Personality Questionnaire (SPQ-B; Raine and Benishay, 1995) (e.g., Dagnall et al., 2017a) and the Unusual Experiences subscale of the Oxford-Liverpool Inventory of Feelings and Experiences scale (O-LIFE; Mason et al., 1995, 2005) (e.g., Dagnall et al., 2016a). This suggests that interactions between positive schizotypal features (i.e., strange perceptual and cognitive sensations and/or magical interpretations) and paranormal belief influence psychological functioning (i.e., well-being) (Dagnall et al., 2010; Dembińska-Krajewska and Rybakowski, 2014). Schizotypy in this context, is allied also to functional deterioration (see Ettinger et al., 2015).

The attraction of schizotypy to researchers is that the construct allows investigation of schizophrenic and psychotic propensities in non-clinical populations without confounds found in schizophrenic patients (cognitive impairment, severe symptoms, etc.) (Barrantes-Vidal et al., 2015). However, research on schizotypy is not straightforward because there is theoretical debate about the relationship between schizotypy and schizophrenia (Barrantes-Vidal et al., 2015). This centres on the fact that models define mental health-illness continuum in different ways. The quasi-dimensional model contends that “true” schizotypy is seen in only a minority of the population (possessing a schizotaxic inheritance), where it manifests as either latent signs or disorder (Grant, 2015; Claridge, 2018). The fully dimensional model views schizotypy as a dimension of personality, whereas schizophrenia is a discrete breakdown process (Grant et al., 2018).

This manuscript adopted the fully dimensional perspective advocated by Claridge (1985), where “schizotypy denotes a range of enduring personality traits, reflected in cognitive style and perceptual experiences, arising from a combination of polygenetic and environmental determinants, which are normally distributed within the general population” (Grant et al., 2018, p. 558). This standpoint concurs with the observation that high levels of schizotypy in non-clinical populations do not inevitably lead to the development of psychopathology (Dembińska-Krajewska and Rybakowski, 2014). This approach is advantageous to the extent that it allows comparisons with commensurate personality and individual differences-oriented research.

### The Present Study

A methodological concern that limits the usefulness of prior research derives from the observation that investigations have typically adopted a variable-centred approach (e.g., correlation based), which regards constructs as independent but related. Although the variable-centred approach provides useful theoretical insights, it is limited since it fails to consider how paranormal belief and psychopathology-related factors (i.e., schizotypy) combine to influence well-being.

Recognising this issue, researchers have used the “person-centred” approach to advance conceptual understanding of paranormal belief (e.g., Goulding, 2005; Schofield et al., 2016). This has involved the use of clustering analysis (CA). CA is a data-driven approach, which begins by randomly assigning cases to a specified number of clusters, and then reassigns cases to minimise the distance to the cluster centre (Gartstein et al., 2017). Although CA provides useful discernments about heterogeneous populations, it possesses limitations (see Flensborg Damholdt et al., 2012). Specifically, repeated runs fail to produce the same results because clusters derive from initial random assignment. Additionally, solutions vary as a function of variable selection, entry order, and scaling. A further constraint is that no statistical assistance is provided to determine cluster numbers within models. This results in inconsistent cluster solutions (see also Terhune and Cardeña, 2010).

Latent approaches are considered superior to CA because they address many of these limitations. Latent profile analysis (LPA) enables the use of fit statistics to assess model selection and permits inclusion of covariates (DiStefano and Kamphaus, 2006). The computation of model fit statistics ensures that class enumeration is less arbitrary (Terhune and Cardeña, 2010; Flensborg Damholdt et al., 2012). Acknowledging this, investigators have recently employed LPA (see Denovan et al., 2018; Drinkwater et al., 2021, 2022).

Latent profile analysis identifies profiles of individuals based on responses to a series of continuous variables (i.e., indicators). LPA assumes that there are unobserved profiles that generate patterns of responses on indicator items. The use of LPA is appropriate when constructs occur simultaneously among populations by virtue of positive correlations (i.e., paranormal, and religious beliefs). Specifically, LPA provides an analytical method that assesses latent heterogeneous patterns and affords a sophisticated appreciation of construct overlap (Wilson et al., 2014).

Illustratively, Denovan et al. (2018) used LPA to identify subtle variations in reasoning performance as a function of paranormal belief and schizotypy. Low levels of paranormal belief were associated with superior performance on perception of randomness (i.e., avoiding the tendency to perceive relatedness within unconnected stimuli; Dagnall et al., 2007a,^2014^) and had no effect on conjunction fallacy (determining whether co-occurring events were more likely to occur than constituent events; Dagnall et al., 2016b,2017b). Furthermore, schizotypy had only a negligible effect on overall reasoning performance. These findings extended previous schizotypy-based research, which identified differences in responses because of cluster (subscale scores, Loughland and Williams, 1997) and profile (schizotypy amalgamated with temperament and character, Hori et al., 2014) membership.

Prior to the Denovan et al. (2018) paper, research had failed to explore how joint paranormal belief and schizotypy profiles influence scores on related factors. Thus, the application of LPA to psychopathological outcomes provides important novel conceptual insights into the cognitive-perceptual factors that affect psychological well-being. Within the current study, to ensure comparability with preceding research, depression, and manic depressiveness were included alongside paranormal belief and schizotypy to produce an amalgamated “Psychopathology” factor.

Well-being was assessed on a range of commonly used health-related outcomes (perceived stress, somatic complaints, and life satisfaction). Collectively, these assess a broad range of psychological and physiological outcomes. Although exploratory in nature, it was anticipated that the presence of high paranormal belief and psychopathology scores would be associated with high levels of perceived stress and somatic complaints, and lower life satisfaction.

## Materials and Methods

### Participants

A sample of 4,402 respondents took part (*M*age = 48.53, *SD* = 15.60, range 18–89). There were 1,913 males (*M*age = 54.13, *SD* = 15.08, range 18–89), 2,473 females (*M*age = 44.25, *SD* = 14.57, range 18–89), 10 non-binary (*M*age = 39.90, *SD* = 16.53, range 19–71), and 6 did not disclose gender (*M*age = 45.50, *SD* = 21.54, range 22–78). Skewness and kurtosis were between −2.0 and +2.0 and deemed acceptable (Byrne, 2010). Recruitment occurred through Bilendi, a recognised supplier of quality, representative online samples (Salak et al., 2021). Bilendi distributed the study link, housed in Qualtrics, to their participant panel. This comprised a United Kingdom-based sample with a minimum age of 18 years and an equal gender split. Bilendi obtains data from recruitment panels, which are derived from a pre-arranged pool of individuals who have consented to respond to surveys in research studies. Data collected in this manner is comparable with traditional, self-managed approaches (Kees et al., 2017).

### Measures

#### Psychopathology-Related Measures

##### Revised Paranormal Belief Scale

The RPBS (Tobacyk, 2004) is a widely used measure of paranormal belief. The instrument comprises 26 statements (e.g., “The number 13 is unlucky”). Participants respond using a seven-point Likert scale (1 = strongly disagree to 7 = strongly agree). Consistent with Rasch, scaling scores were converted to 0–6 (see Irwin, 2009). Higher scores indicate greater belief in the paranormal. The RPBS has established psychometric properties (i.e., validity and reliability) (Drinkwater et al., 2017). In this study, the RPBS demonstrated excellent omega (ω = 0.96) and alpha (α = 0.95) reliability.

##### The Oxford-Liverpool Inventory of Feelings and Experiences

The O-LIFEshort (Mason et al., 2005) is an abridged (43-items) version of the 104-item O-LIFE (Mason et al., 1995), which assesses schizotypal traits in non-clinical samples. The instrument comprises four subscales: Unusual Experiences (12-items), Cognitive Disorganisation (11-items), Introvertive Anhedonia (10-items), and Impulsive Non-Conformity (10-items). Unusual Experiences examines positive schizotypy (perceptual aberrations, magical thinking, and hallucinations). Cognitive Disorganisation measures disorganised elements of psychosis (e.g., poor attention/concentration). Introvertive Anhedonia assesses negative schizotypy features including withdrawal and avoidance of intimacy. Impulsive Non-conformity reflects lack of self-control (impulsive and antisocial behaviour).

Items are presented in question form (e.g., “Do you feel very close to your friends?”) and participants respond using a binary response format (No/Yes). The overall scale has high internal consistency, with alphas ranging from 0.62 to 0.80 among its subscales (Mason et al., 2005). Reliability in this study was good for Unusual Experiences (ω = 0.85, α = 0.95) and Cognitive Disorganisation (ω = 0.85, α = 0.85). Lower estimates were observed for Introvertive Anhedonia (ω = 0.61, α = 0.61) and Impulsive Non-Conformity (ω = 0.64, α = 0.64). However, these are consistent with previous research using this measure (see Mason et al., 2005).

##### Center for Epidemiologic Studies-Depression Scale

The CES-D (Radloff, 1977) is a widely used measure of depressive symptoms containing 20-items with a response scale of 0 (rarely) to 3 (most or all of the time). Items (e.g., “I felt lonely”) focus on the past week. High internal reliability exists (Hann et al., 1999). The current study observed excellent reliability (ω = 0.93, α = 0.93).

##### Manic-Depressiveness Scale

Thalbourne et al. (1994) developed the Manic-Depressiveness Scale (MDS), which includes two 9-item True/False subscales. One subscale assesses manic experience (e.g., “I have been through times when it seemed almost unnecessary for me to eat”), and the other captures depressive experience (e.g., “I tend to sleep more when life is going badly”). The scale has been utilised frequently within paranormal belief research. Psychometrically, the MDS has established reliability and validity (see Lester, 2000). Within this study, both the Depressive Experience scale (ω = 0.79, α = 0.79), and the Manic Experience scale (ω = 0.67, α = 0.62) were consistent with Lester (2000).

#### Well-Being

##### The 10-Item Perceived Stress Scale

The PSS-10 (Cohen and Williamson, 1988) assesses the degree of uncontrollability and unpredictability stress present in an individual’s life. The measure has 10-items (e.g., “how often have you felt nervous and “stressed”?”), which focus on the past month from 0 (never) to 4 (very often). The PSS-10 has established psychometric properties (Denovan et al., 2019). In this study, good reliability was observed (ω = 0.86, α = 0.86).

##### The Somatic Symptom Scale-8

The SSS-8 assesses sensitivity to somatic complaints by focusing on a 7-day period (Gierk et al., 2014). Items are presented as somatic symptom burdens (e.g., “chest pain or shortness of breath”) and participants respond *via* a five-point scale from 0 (not at all) to 4 (very much). Gierk et al. (2014) reported good alpha reliability, which was also found in this study (ω = 0.89, α = 0.89).

##### The Satisfaction With Life Scale

The SWLS (Diener et al., 1985) measures global cognitive judgements of life satisfaction as an index of subjective well-being. It comprised five-items (e.g., “my life is close to ideal”) rated on a seven-point Likert scale (1 strongly disagree to 7 strongly agree). Internal consistency is high (Diener et al., 1985). This was also the case in the current study (ω = 0.93, α = 0.92).

### Procedure

Participants retrieved study information by clicking on a web link. After reading the information sheet, only participants meeting the inclusion criteria and providing informed consent proceeded to the survey. This comprised a section on demographics (age and preferred gender), and measures. Participants were encouraged to carefully read all items and told that there were no correct answers and that they should work at their own pace. Questionnaire order rotated to limit possible order effects. All respondents were debriefed after completing the questionnaire.

The study was cross-sectional, data was collected at one point in time. A criticism of this approach is that it can produce spurious variance, where similarity between variables arises from the common method rather than the constructs under observation (Spector, 2019). To reduce the potential for this bias the researchers employed procedural countermeasures. These took the form of specific instructions, which created psychological distance between measures by emphasising dissimilarities between scales and constructs. Instructions also attempted to reduce social desirability and evaluation apprehension by emphasising that were no right answers and that responses should reflect personal thoughts and preferences (Krishnaveni and Deepa, 2013).

### Analysis

Preliminary analysis assessed descriptive relationships. Latent profile analysis (LPA) based on Paranormal Belief and psychopathology scores (Schizotypy, Depression, and Manic Depressiveness) then determined group membership. Analysis used Mplus version 7 (Muthén and Muthén, 2012).

Model fit evaluated solutions with increasing numbers of latent profiles (beginning with a 1-profile model) until the inclusion of additional profiles was no longer justified. Several indices determined the optimal number of sub-groups: Akaike Information Criterion (AIC; Akaike, 1987), Bayesian Information Criterion (BIC; Schwarz, 1978), sample-size adjusted BIC (ssaBIC; Sclove, 1987), Lo-Mendell-Rubin-adjusted likelihood ratio test (LMR-A-LRT; Lo et al., 2001), and entropy (Ramaswamy et al., 1993). Lower AIC, BIC, and ssaBIC suggested superior fit. LMR-A-LRT tested fit using the 0.01 significance level, and entropy scores > 0.8 indicated comprehensive profile separation relative to data (Ramaswamy et al., 1993). Lastly, an assessment of profile differences occurred in relation to well-being measures.

## Results

### Descriptive Statistics

Data screening identified five outliers (i.e., data points with z-scores greater than 3.25) and were transformed to the next highest score (Tabachnick and Fidell, 2001). This manuscript utilised Cohen’s (1988; 1992) guidelines for correlation magnitude (i.e., 0.10 = small, 0.30 = medium, and 0.50 = large). Gignac and Szodorai (2016), however, contend that these are too exigent because they stem from qualitative impression, rather than quantitative analysis of data. They recommend correlations of 0.10, 0.20, and 0.30 as small, typical, and large. Interpreting effect sizes *via* these classifications suggests more meaningful relationships exist among observed relationships.

Small to large correlations existed between schizotypy (Unusual Experiences, Cognitive Disorganisation, Impulsive Non-Conformity), Paranormal Belief, Depression, Depressive Experience, and Manic Experience (Table 1). Introvertive Anhedonia, however, demonstrated weaker relationships. Well-being variables (Perceived Stress, Somatic Complaints, Life Satisfaction) demonstrated weak to large relationships with Paranormal Belief and psychopathology-related variables. The exception to this was the non-significant association between Paranormal Belief and Life Satisfaction.

**TABLE 1:** Descriptive statistics and intercorrelations among all study variables.

Variable	Scaled mean	*SD*	1	2	3	4	5	6	7	8	9	10	11
1. Paranormal belief	2.20	1.23		0.55**	0.30**	0.03	0.28**	0.30**	0.25**	0.28**	0.24**	0.31**	−0.01
2. Unusual experiences	0.31	0.27			0.58**	0.06**	0.48**	0.43**	0.51**	0.51**	0.35**	0.41**	−0.10**
3. Cognitive disorganisation	0.39	0.31				0.23**	0.54**	0.52**	0.53**	0.60**	0.54**	0.46**	−0.30**
4. Introvertive anhedonia	0.38	0.23					0.20**	0.17**	0.10**	0.23**	0.25**	0.23**	−0.29**
5. Impulsive non-conformity	0.26	0.21						0.48**	0.47**	0.58**	0.48**	0.42**	−0.29**
6. Depression	0.96	0.49							0.47**	0.57**	0.56**	0.60**	−0.27**
7. Manic experience	1.37	0.26								0.70**	0.38**	0.40**	−0.17**
8. Depressive experience	1.33	0.27									0.56**	0.52**	−0.36**
9. Perceived stress	1.74	0.78										0.53**	−0.55**
10. Somatic complaints	2.14	0.90											−0.28**
11. Life satisfaction	4.08	1.47											

**Indicates p < 0.001.

### Latent Profile Analysis

A preliminary appraisal of 1 vs. 2-profile solutions indicated better fit for the 2-profile model, evident from lower AIC, BIC and ssaBIC alongside significant LMR-A-LRT (Table 2). Subsequently, appraisal of 2 vs. 3-profile models suggested greater fit for the 3-profile solution, and comparison of 3 and 4-profile models supported the 4-profile conceptualisation. Lastly, a 5-profile solution revealed a non-significant improvement, LMR-A-LRT *p* = 0.012. The 4-profile model was chosen to be superior because, though larger AIC, BIC and ssaBIC occurred vs. the 5-profile model, the 5-profile approach indicated non-significant improvement alongside lower entropy (0.82 vs. 0.83).

**TABLE 2:** Fit of competing latent profile models.

Model	AIC	BIC	ssaBIC	LMR-A	LMR-A *p-*value	Entropy
1-class	24477.47	24579.71	24528.87			
2-class	14198.49	14358.25	14278.81	10162.40	<0.001	0.88
3-class	11951.72	12168.99	12060.95	2235.17	<0.001	0.84
4-class	11137.03	11411.82	11275.18	821.80	<0.001	0.83
5-class	10487.82	10820.11	10654.88	658.49	0.012	0.82

*AIC, Akaike Information Criterion; BIC, Bayesian Information Criterion; ssaBIC, sample-size adjusted BIC; LMR-A, Lo-Mendell-Rubin-adjusted likelihood ratio test.*

**FIGURE 1:**
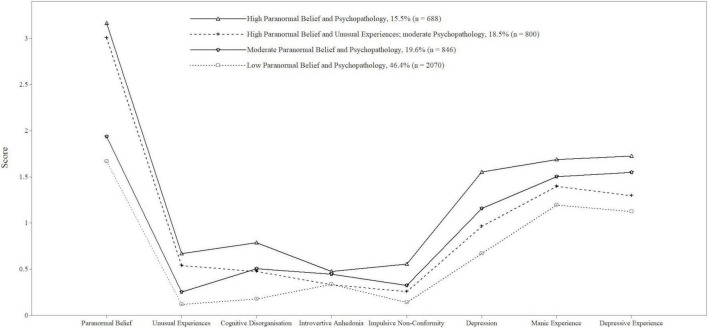
Pattern of scaled mean scores for paranormal belief, schizotypy, depression, and manic depressiveness as a function of latent profile. Profile 1 = high paranormal belief and psychopathology; Profile 2 = high paranormal belief and unusual experiences; moderate psychopathology; profile 3 = moderate paranormal belief and psychopathology; Profile 4 = low paranormal belief and psychopathology.

The 4-profile model (Figure 1) depicts profiles/classes from higher to lower scores. Profile 1 (labelled as “high Paranormal Belief and Psychopathology”) comprised 15.5% (*n* = 688) of the sample and reflected higher scores among all variables but Introvertive Anhedonia. Profile 2 (identified as “high Paranormal Belief and Unusual Experiences; moderate Psychopathology”) included 18.5% (*n* = 800) and evidenced high Paranormal Belief and Unusual Experiences relative to scaled means, and moderate scores on the remaining variables. Moderate in terms of scoring similarly to scaled means. Profile 3 (“moderate Paranormal Belief and Psychopathology”) comprised 19.6% (*n* = 846) and overlapped with Profile 2 in terms of possessing lower Paranormal Belief and Unusual Experiences, but slightly higher scores for the other variables. The scores were close to scaled means to qualify as moderate, however. Profile 4 (“low Paranormal Belief and Psychopathology”) included 46.4% (*n* = 2070) and evidenced lower scores across variables. Average latent class probabilities suggested good discrimination (Profile 1 = 0.95, Profile 2 = 0.92, Profile 3 = 0.84, Profile 4 = 0.83).

The four subgroups reflected low through moderate to high levels of Paranormal Belief and Psychopathology. These Profiles were conceptually sound since psychopathology-related measures (i.e., Depression, Manic Experience, and Depressive Experience) correlated moderately with Paranormal Belief, and more highly with each other. These intra psychopathology-related measure associations, however, were not indicative of multicollinearity (i.e., less than 0.8; Tabachnick and Fidell, 2001).

### Assessment of Latent Profiles in Relation to Perceived Stress, Somatic Complaints, and Life Satisfaction

A multivariate analysis of variance (MANOVA) scrutinised relationships between latent profiles and Perceived Stress, Somatic Complaints, and Life Satisfaction (Table 3). A significant main effect of group existed, Pillai’s trace = 0.43, *F*_(9,13200)_ = 249.61, *p* < 0.001, η^2^ = 0.15 (large effect size). Significant effects of group occurred concerning Perceived Stress, Somatic Complaints, and Life Satisfaction.

**TABLE 3:** The effects of group (latent profile) in relation to perceived stress, somatic complaints, and life satisfaction.

	Dependent variable					
	
	Perceived stress	Somatic complaints	Life satisfaction			
	
	ANOVA			MANOVA		
		
	*F^df^* (*Sig.*; η^2^)	*F^df^* (*Sig.*; η^2^)	*F^df^* (*Sig.*; η^2^)	Pillai’s trace	*F^df^* (*Sig.*)	η^2^
**Variable**						
Group	705.82^3,4400^ (<0.001; 0.33)	647.53^3,4400^ (<0.001; 0.31)	194.26^3,4400^ (<0.001; 0.12)	0.43	249.61^9,13200^ (<0.001)	0.15
	
	**Pairwise comparisons (scaled mean differences) between classes**					
	
**Class contrast**	**Mean diff. (*Sig.*)**	**Mean diff. (*Sig.*)**	**Mean diff. (*Sig.*)**			

Profile 1 vs. Profile 2	0.66 (<0.001)	0.88 (<0.001)	−0.70 (<0.001)			
Profile 1 vs. Profile 3	0.34 (<0.001)	0.67 (<0.001)	0.09 (1.00)			
Profile 1 vs. Profile 4	1.16 (<0.001)	1.39 (<0.001)	−1.07 (<0.001)			
Profile 2 vs. Profile 3	−0.31 (<0.001)	−0.21 (<0.001)	0.79 (<0.001)			
Profile 2 vs. Profile 4	0.50 (<0.001)	0.51 (<0.001)	−0.37 (<0.001)			
Profile 3 vs. Profile 4	0.81 (<0.001)	0.72 (<0.001)	−1.16 (<0.001)			

*Profile 1 = high paranormal belief and psychopathology (n = 688); Profile 2 = high paranormal belief and unusual experiences; moderate psychopathology (n = 800); Profile 3 = moderate paranormal belief and psychopathology (n = 846); Profile 4 = low paranormal belief and psychopathology (n = 2070).*

*Post hoc* mean contrasts (using Bonferroni correction; Table 3) revealed that Profile 1 exhibited significantly greater Perceived Stress, Somatic Complaints, and lower Life Satisfaction than the other subgroups (Profiles 2 to 4) aside from Profile 3 on Life Satisfaction. Profile 3 scored higher on Perceived Stress, Somatic Complaints, and lower on Life Satisfaction then Profile 2 and Profile 4. Lastly, Profile 2 reported greater Perceived Stress, Somatic Complaints, and lower Life Satisfaction than Profile 4.

## Discussion

Emergent subgroups reflected subtle variations in paranormal belief and psychopathology, which were associated with differences on well-being measures. Specifically, Profile 1 (high Paranormal Belief and Psychopathology) indexed lower well-being in comparison with the other profiles (Profile 2–4). Contrastingly, Profile 4 (low Paranormal Belief and Psychopathology) evidenced greater well-being vs. the other profiles (Profiles 1–3). Profile 3 (moderate Paranormal Belief and Psychopathology) indexed lower well-being than Profile 2 (high Paranormal Belief and Unusual Experiences; moderate Psychopathology), suggesting that belief in the paranormal is not necessarily contributory to psychological adjustment. Additionally, results indicated that believers are a heterogenous rather than homogeneous population.

Zero-order correlations were consistent with preceding research. Paranormal Belief demonstrated a similar pattern of associations with O-LIFEshort subscales to those reported by Dagnall et al. (2016c). Particularly, Paranormal Belief was most strongly related to Unusual Experiences, correlated with Cognitive Disorganisation and Impulsive Non-conformity, but was not significantly associated with Introvertive Anhedonia. These outcomes correspond to general dimensional models of schizotypy (Kwapil et al., 2008). For instance, they are consistent with the distinction between positive (i.e., unusual experiences, perceptions, beliefs, and magical thinking) and negative (i.e., withdrawal and attenuated ability to experience pleasure) factors.

The positive association between Introvertive Anhedonia and Paranormal Belief is explained by the fact that negative features reflect the tendency to gain less satisfaction from engaging in effortful and deliberative thought (Broyd et al., 2019). Thus, in comparison to positive schizotypy, which is associated with the production of unusual experiences, perceptions, beliefs and magical thinking, negative schizotypy is less cognitive (Broyd et al., 2019). This, in part, explains why positive characteristics are conducive to the generation and maintenance of paranormal beliefs, whereas negative features are unlikely to directly influence supernatural credence. Future research is required to assess the extent to which differences in cognitive engagement influence belief in the paranormal.

Examination of profiles indicated that belief and psychopathological factors interacted in complex ways. Respondents high in Paranormal Belief were differentiated by elevated global (Profile 1) vs. specific (Unusual Experiences) (Profile 2) Psychopathology scores. The presence of a profile characterised by high Unusual Experiences aligns with Loughland and Williams (1997). The Unusual Experiences subscale reflects mainly positive schizotypal characteristics such as perceptual distortions and magical thinking, which align with the reality distortion syndrome of positive schizophrenic symptoms (Liddle, 1987; Loughland and Williams, 1997). Perceptual distortions represent an attenuated form of hallucination, and magical thinking signifies weaker type delusional thoughts.

In the present study, Profile 2 attributes were associated with higher levels of well-being than the global high (Profile 1) and moderate psychological adjustment (Profile 3) subgroups. This suggests that high Paranormal Belief is not necessarily concomitant with lower psychological adjustment and reduced well-being. Although, caution is required when drawing comparison with Loughland and Williams (1997), since they used agglomerative hierarchical clustering rather than LPA, and their analysis considered only schizotypy.

Despite this caveat, the presence of differing high belief profiles has important implications for subsequent research as they are differentially associated with well-being. The presence of a Paranormal sub-group with relatively low Psychopathology scores is consistent with the high levels of supernatural endorsement observed in general populations. It also aligned with the notion that paranormal beliefs in non-clinical samples represent non-psychotic delusions (Irwin et al., 2012a,b). In this context, beliefs often arise from reality testing deficits where individuals fail to adequately assess the validity of propositions and the evidence from which they derive (Dagnall et al., 2015; Drinkwater et al., 2020). Thus, beliefs alone reflect thinking style preferences rather than variations in psycholopathology.

Using LPA to study paranormal belief and psychopathology is conceptually significant because the method recognises that individuals because of life history vary on both constructs. This is important as paranormal belief and psychopathology may concurrently influence psychological adjustment and well-being. Hence, identifying differing profiles advances knowledge in terms of appreciating how specific combinations of paranormal belief and psychopathology relate to well-being. In this instance, demonstrating that although higher Paranormal Belief and psychopathology generally relate to lower well-being, high Paranormal Belief is not inevitably attendant with poorer psychological functioning and lower well-being.

This conclusion is consistent with related work postulating the existence of happy or benign schizotypes. That is, individuals who experience psychotic-like experiences as rewarding and enhancing. These are individuals, who (in relation to the population means) score extremely high on the positive characteristics, but below average on negative and cognitive/disorganised factors (see Claridge, 2018; Grant and Hennig, 2020).

### Limitations

A limitation concerns the relative distributions of Paranormal Belief and psychopathology-related scores. Explicitly, Paranormal Belief exhibited greater variation compared with psychopathology measures such as schizotypy. Though schizotypy sum totals were analogous to established norms (Mason et al., 2005), range restriction existed because participants came from a non-clinical, general population. In addition, differences existed as a function of the number of items per measure (e.g., Paranormal Belief 26-items vs. Unusual Experiences 12-items). While scaled means were utilised to minimise this (which is advocated with LPA; Uckelstam et al., 2019), high scores on variables should be interpreted as relative rather than absolute.

Moreover, recoding continuous data to create meaningful profiles can lead to information loss (Lanza and Rhoades, 2013). The profiles in this study were statistically and conceptually meaningful, however, it is necessary to guard against reification. Particularly, LPA profiles relate to heterogeneity across a model’s variables, not subtypes of individuals in the population (Lanza and Rhoades, 2013). Too few or too many profiles can be identified through LPA, and it would be valuable for subsequent research to corroborate the current findings by replication and cross-validation (Collins et al., 1994).

## Data Availability Statement

The raw data supporting the conclusions of this article will be made available by the authors, without undue reservation.

## Ethics Statement

Ethical approval was granted by the Manchester Metropolitan University Faculty of Health, Psychology and Social Care Ethics Committee (December 2020; Project ID, 25390). Written informed consent for participation was not required for this study in accordance with the national legislation and the institutional requirements.

## Author Contributions

AD and ND designed the study. AD researched and collated the measures, organised the data collection, and conducted the all analyses. ND provided the conceptual input, summarised the findings, and synthesised the content for all sections. KD and AD edited the final manuscript and prepared the draft for submission. All authors contributed to the article and approved the submitted version.

## Conflict of Interest

The authors declare that the research was conducted in the absence of any commercial or financial relationships that could be construed as a potential conflict of interest.

## Publisher’s Note

All claims expressed in this article are solely those of the authors and do not necessarily represent those of their affiliated organizations, or those of the publisher, the editors and the reviewers. Any product that may be evaluated in this article, or claim that may be made by its manufacturer, is not guaranteed or endorsed by the publisher.
